# Binary solvent participation in crystals of a multi-aromatic 1,2,3-triazole

**DOI:** 10.1107/S2056989024011915

**Published:** 2025-01-01

**Authors:** Jonathan Filley

**Affiliations:** a Oligometrics, Inc., 2510 47th Street, Suite 208, Boulder, CO, 80301, USA; University of Missouri-Columbia, USA

**Keywords:** crystal structure, click chemistry, three-center hydrogen bond

## Abstract

The X-ray crystal structure of a multi-aromatic substituted 1,2,3-triazole is presented, which shows an extensive three-dimensional hydrogen-bonding network involving two water mol­ecules and two aceto­nitrile mol­ecules. The structure of 4-{[(4-{[1-({[(3,4-di­meth­oxy­phen­yl)meth­yl](3-acetamido­phen­yl)carbamo­yl}meth­yl)-1*H*-1,2,3-triazol-4-yl]meth­oxy}-3-meth­oxy­phen­yl)meth­yl]amino}­benzoic acid–aceto­nitrile–water (1/2/2), C_37_H_38_N_6_O_8_·2C_2_H_3_N·2H_2_O, features amine-linked aromatic groups that have a variety functionality including a carb­oxy­lic acid, an acetamido group, and meth­oxy ethers. All *X*—H groups, and seven out of ten heteroatoms with available lone-pair electrons, participate in hydrogen bonding, with the aid of dimer-bridging water mol­ecules and aceto­nitrile mol­ecules whose methyl groups form close contacts with oxygen atoms. The triazole itself is a dimer made using click chemistry from readily available and inexpensive starting materials and is a precursor to larger oligomers, as well as to compounds with a wide array of readily manipulated functionality.

## Abstract 2

Crystals of a multi-aromatic substituted 1,2,3-triazole have an extensive hydrogen-bonding network with two water and two aceto­nitrile mol­ecules participating in the structure. The compound is a dimer serving as a starting material for higher oligomers and mol­ecules featuring extensive functionality.

## 1. Chemical context

Foldamers, or folding oligomers, are synthetic organic mol­ecules that have a propensity to form weak intra­molecular inter­actions and have secondary structure analogous to biomacromolecules (Hill *et al.*, 2001 ▸; Gellman, 1998 ▸). With the goal of preparing functional foldamers, we prepared an unusual dimer mol­ecule that crystallized easily from a mixture of aceto­nitrile and water (compound **1**). An X-ray structure determination was undertaken to understand whether or not its aromatic components stack intra­molecularly (*i.e.*, fold). While the mol­ecule adopts an extended conformation in the crystal, and therefore may be unlikely to form a folding nucleus for larger oligomers based upon it, certain features of the mol­ecule and its inter­actions with solvent mol­ecules are reported here.

The linking chemistry used to make the title compound is the copper-catalyzed alkyne–azide cyclo­addition reaction, also known as the CuAAC click reaction (Kolb *et al.*, 2001 ▸). It is composed of an aromatic 1,2,3-triazole substituted at the 1 and 4 positions with groups that each feature two aromatic rings bearing a variety of functionality. The main criteria for linking these groups are the ready availability and low cost of the starting materials, which are transformed into the moieties indicated with brackets, and are: *A*: 3-acetamido­aniline, *B*: 3,4-di­meth­oxy­benzaldehye, *C*: vanillin, and *D*: ethyl 4-amino­benzoate. The starting materials were also chosen for their functionality, and the ease with which they can be modified. For instance, the acetamido group in *A* could be changed to a longer chain amide (or an amide containing additional functionality), the 4-meth­oxy group in *B* can be changed to other ethers (including ones featuring alkynes) using vanillin as the starting material, the meth­oxy group in *C* can easily be obtained as an eth­oxy group, which could be a useful spectroscopic handle, and leaving the carb­oxy­lic acid in *D* as an ester decreases its polarity. While the main objective for making the title compound was to use it as a starting point for making larger oligomers, it is also possible to envision it for preparing a large variety of mol­ecules with a wide range of functionality.
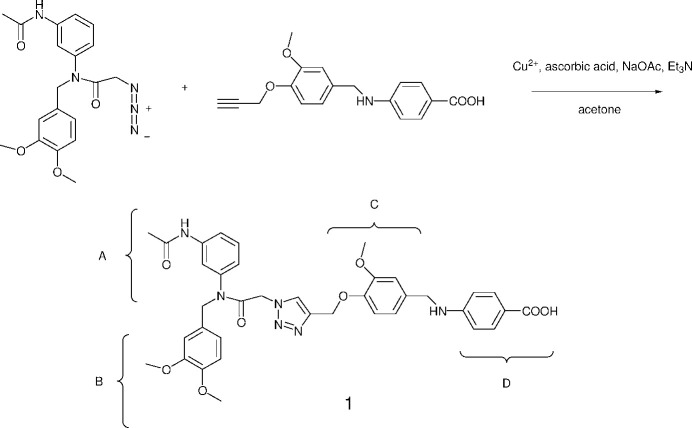


## 2. Structural commentary

**Figure 1:**
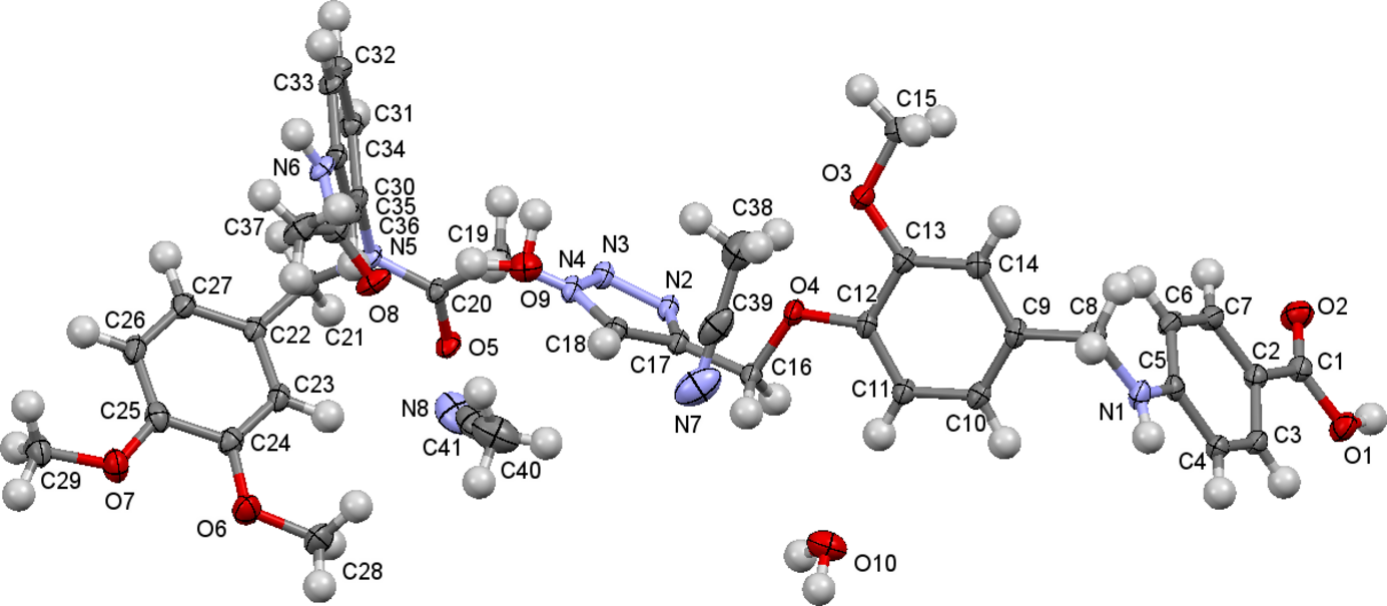
A view of the title compound with two water and two aceto­nitrile mol­ecules as solvents of crystallization. Atoms are displayed as ellipsoids at the 50% probability displacement level.

The extended conformation is shown in Fig. 1 ▸, which also shows two water mol­ecules and two aceto­nitrile mol­ecules as solvents of crystallization. The mol­ecular structure confirms the cyclo­addition reaction proceeds with the expected regiochemistry to give a 1,4-substituted 1,2,3-triazole. The amine nitro­gen atom is essentially planar [C6—C5—N1—C8 torsion angle = 0.0 (2)°], indicating the lone pair electrons are conjugated with the aromatic ring, facilitated by the electron withdrawing carb­oxy­lic acid, while the aromatic ring attached at the amide nitro­gen atom is essentially non-planar [C20—N5—C30—C31 torsion angle = 107.1 (2)°] indicating the amide lone pair is not conjugated to the aromatic ring.

## 3. Supra­molecular features

**Figure 2:**
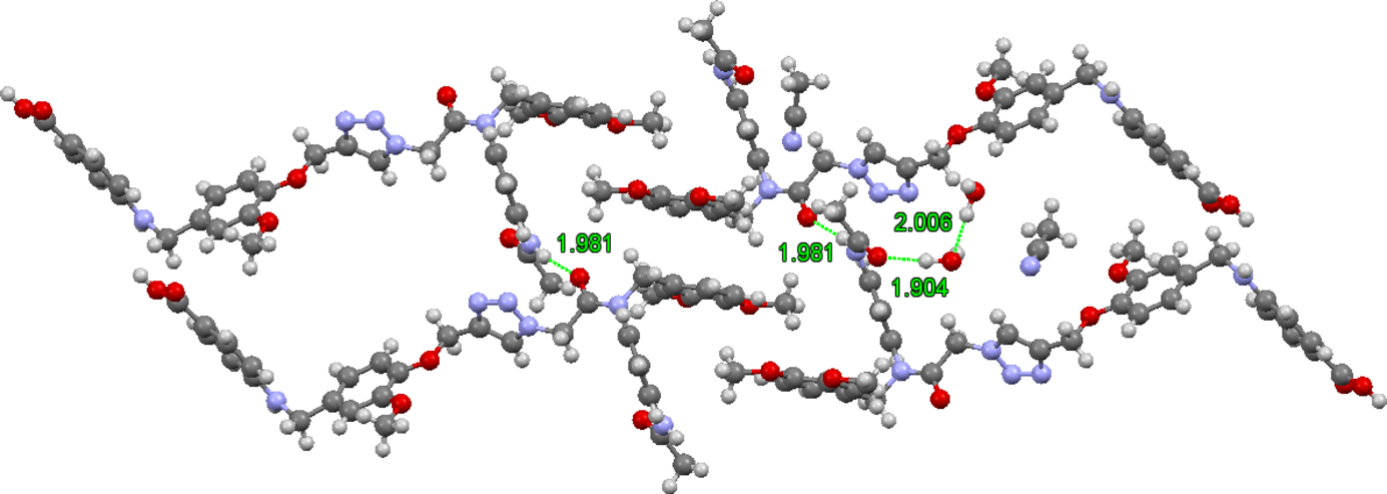
Four mol­ecules highlighting the acetamido–amide hydrogen bond, the water dimer bound to the acetamide carbonyl group, π-stacking, and the solvents of crystallization.

The crystal packing in Fig. 2 ▸ shows four mol­ecules with solvent mol­ecules included on the right side of the image. It can be seen that an aceto­nitrile mol­ecule helps fill the void space between two alk­oxy benzenes, the water mol­ecules are hydrogen bonded together [2.01 (2) Å, see Table 1 ▸], and one mol­ecule of this pair is hydrogen bonded to an acetamide carbonyl [1.90 (3) Å]. The amide hydrogen atom of this acetamide is hydrogen bonded [1.98 (2) Å] to the linking amide adjacent to the triazole of the next mol­ecule in the crystal. The dimeth­oxy benzenes are π-stacked in a face-to-face manner on a pairwise basis, with the local dipoles oriented anti­parallel, and with the methyl­ene carbon atom (C21) positioned almost directly over an ether oxygen atom (O7). The second pair of stacked dimeth­oxy benzenes is offset several bond lengths such that a methyl group (C29) is located almost directly over a benzene ring carbon (C27).

**Figure 3:**
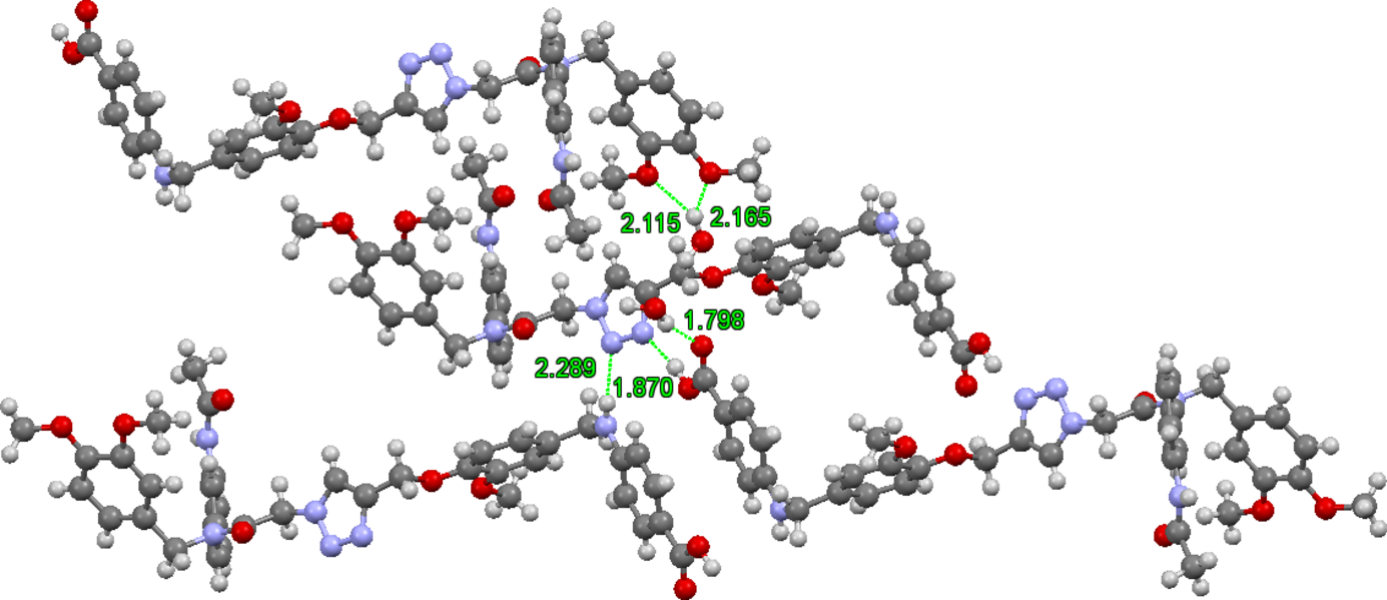
Four mol­ecules showing hydrogen bonding to the 1,2,3-triazole, water hydrogen bonding to the carb­oxy­lic acid carbonyl group, and water participating in the three-center hydrogen bond.

Fig. 3 ▸ shows four mol­ecules focusing on the hydrogen bonding to the triazole, as well as the bridge hydrogen bonding facilitated by the two water mol­ecules. The carb­oxy­lic acid hydrogen bonds to the 3-position of the triazole [1.87 (3) Å] and the amine NH forms a hydrogen bond to the 2-position of the triazole [2.29 (2) Å]. The carb­oxy­lic acid serves as an hydrogen-bond acceptor from a water mol­ecule [1.80 (3) Å]. This water mol­ecule forms the hydrogen bond noted above to the other water mol­ecule, which forms a three-center hydrogen bond to the dimeth­oxy benzene of the fourth mol­ecule displayed [2.12 (3), 2.17 (3) Å]. In total, seven of the ten heteroatoms of the title compound with available lone-pair electrons behave as hydrogen-bond acceptors, creating an extensive three-dimensional hydrogen-bonded network. The dimeth­oxy benzene π-stacking in Fig. 2 ▸ suggests mol­ecules with multi-aromatic side chains bearing dimeth­oxy benzene units could encourage folding.

**Figure 4:**
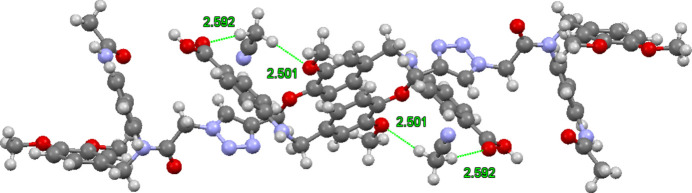
An inter­twined dimer showing close contacts between an aceto­nitrile mol­ecule and oxygen atoms.

**Figure 5:**
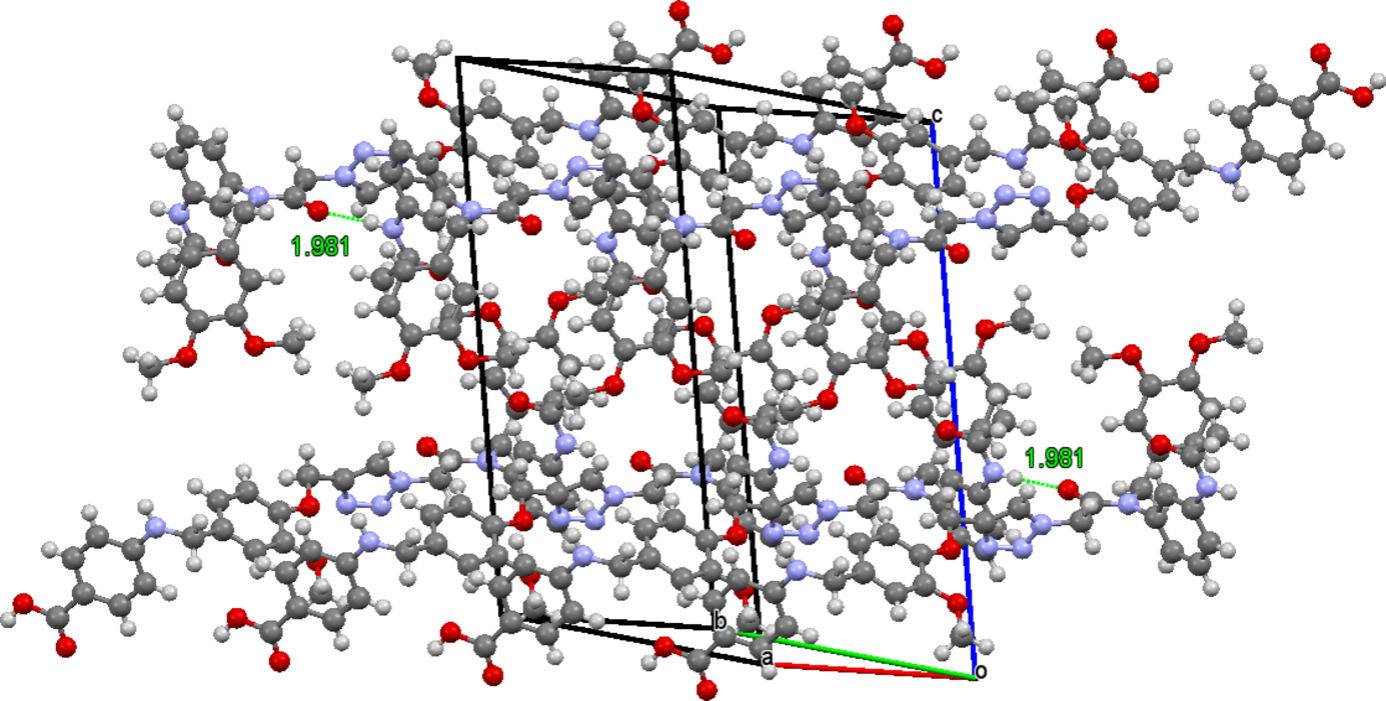
Unit cell superimposed on two sets of hydrogen bonded tetra­mers, showing the amide hydrogen bonding.

The close contacts between the C38 hydrogen atoms of an aceto­nitrile mol­ecule and oxygen atoms are highlighted in Fig. 4 ▸. The oxygen atoms are O6 of the dialk­oxy benzene unit [2.50 (3) Å], and O2 of the carb­oxy­lic acid [2.59 (3) Å]. Two mol­ecules are inter­twined about the dialk­oxy benzene units with symmetrically disposed aceto­nitrile mol­ecules. Fig. 5 ▸ shows the unit cell superimposed on amide-to-amide hydrogen bonded tetra­mers with the solvent mol­ecules omitted, with the top unit oriented with N1, N2, and N3 of the triazoles facing the viewer, and the same atoms of the bottom unit facing away from the viewer. Images of mol­ecular structures were manipulated using *Mercury* (Macrae *et al.*, 2020 ▸).

## 4. Database survey

Since crystalline carb­oxy­lic acids have a tendency to form dimers analogous to the well-known acetic acid dimer, as in the case of 5-bromo-2-(phenyl­amino)­benzoic acid (Kang & Long, 2024 ▸), it is notable that the carb­oxy­lic acid of the title compound forms a hydrogen bond to the triazole. Carb­oxy­lic acids hydrogen bonded to heterocyclic nitro­gen atoms (Ladraa *et al.*, 2010 ▸) and to the 3-position of 1,2,3-triazoles (Lin, 2010 ▸) have been examined. A 1,2,3-triazole click reaction product has also been structurally characterized (Zukerman-Schpector *et al.*, 2017 ▸). A similar arrangement to the π-stacking with dipoles aligned anti-parallel seen for the dimeth­oxy benzene units has been observed in 2-meth­oxy-5-nitro­aniline (Filley, 2024 ▸).

## 5. Synthesis and crystallization

The azide and alkyne in the scheme were prepared in seven steps as detailed in the supporting information. Reductive aminations were performed according to Touchette (2006 ▸). The title compound was made as follows: A 50 ml round-bottomed flask was charged with 0.767 g (2.00 mmole) of *N*-(3-acetamido­phen­yl)-*N*-(3,4-di­meth­oxy­benz­yl)azido­acetamide, 0.623 g (2.00 mmole) of 4-(4-proparg­yloxy-3-meth­oxy­benzyl­amino)­benzoic acid, 0.28 ml (2.0 mmole) of tri­ethyl­amine and 20 ml of acetone. The solids were dissolved with stirring and treated with 2.0 ml of an aqueous solution containing a mixture of 0.1 *M* ascorbic acid and 0.1 *M* sodium acetate, the buffered mixture was then treated with 0.4 ml of aqueous 0.1 *M* CuSO_4_. The flask was flushed with N_2_, capped with a septum, and stirred overnight. The resulting inhomogeneous mixture was poured into 100 ml of water containing 15 mg of Na_2_EDTA and 300 mg of NaOH, giving a clear solution. When this solution was acidified with 5 ml of 6*M* HCl, a gooey precipitate resulted, which was isolated by deca­nting off the aqueous solution, boiling in 10 ml of ethanol, and pouring the hot ethanol solution into 100 ml of water followed by about 15 ml of saturated NaCl. The resultant precipitate was filtered off and allowed to air dry, 1.23 g (88%) snow white powder. ^1^H NMR (400 MHz, CD_3_CN/1% D_2_0): 2.04 (*s*, 3H), 3.70 (*s*, 3H), 3.73 (*s*, 3H), 3.74 (*s*, 3H), 4.27 (*s*, 2H), 4.80 (*s*, 2H), 4.96 (*s*, 2H), 5.02 (*s*, 2H), 6.60–7.75 (*m*, 14H), 7.84 (*s*, 1H). ^13^C NMR (CD_3_CN/1% D_2_0): 23.3, 46.2, 51.5, 52.6, 55.1, 55.2, 55.3, 61.9, 111.2, 111.4, 111.6, 112.0, 113.6, 117.3, 117.5, 119.1, 119.2, 119.3, 121.0, 123.4, 125.9, 129.2, 130.1, 131.5, 132.5, 140.2, 140.6, 143.1, 146.7, 148.5, 149.0, 149.5, 152.5, 165.4, 167.7, 169.3. X-ray quality crystals can be obtained by dissolving 20 mg of the product in 0.8 ml of aceto­nitrile + 1 drop water, followed by the addition of 0.8 ml additional water, and setting aside for several days at room temperature. The solvents of crystallization should be preserved by not allowing the crystals to dry out.

## 6. Refinement

Crystal data, data collection and structure refinement details are summarized in Table 2 All H-atom parameters were refined. ▸

## Supplementary Material

Crystal structure: contains datablock(s) I. DOI: 10.1107/S2056989024011915/ev2012sup1.cif

Structure factors: contains datablock(s) I. DOI: 10.1107/S2056989024011915/ev2012Isup4.hkl

Supporting information file. DOI: 10.1107/S2056989024011915/ev2012sup3.doc

Supporting information file. DOI: 10.1107/S2056989024011915/ev2012Isup4.cml

CCDC reference: 2408502

Additional supporting information:  crystallographic information; 3D view; checkCIF report

## Figures and Tables

**Table 1:** Hydrogen-bond geometry (Å, °)

*D*—H⋯*A*	*D*—H	H⋯*A*	*D*⋯*A*	*D*—H⋯*A*
O9—H9*A*⋯O2^i^	0.96 (3)	1.80 (3)	2.7173 (17)	160 (2)
O9—H9*B*⋯O8	0.99 (3)	1.90 (3)	2.8610 (18)	164 (2)
O10—H10*A*⋯O6^ii^	0.96 (3)	2.12 (3)	2.9441 (17)	144 (2)
O10—H10*A*⋯O7^ii^	0.96 (3)	2.17 (3)	2.9928 (17)	143 (2)
O10—H10*B*⋯O9^iii^	0.95 (3)	2.00 (3)	2.9346 (19)	167 (2)
N1—H1*A*⋯N3^iv^	0.90 (2)	2.29 (2)	3.1834 (17)	174.6 (18)
N6—H6⋯O5^v^	0.85 (2)	1.98 (2)	2.8200 (15)	168.6 (19)
O1—H1⋯N2^vi^	0.91 (3)	1.87 (3)	2.7614 (16)	166 (3)

Symmetry codes: (i) 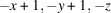; (ii) 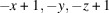; (iii) 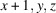; (iv) 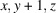; (v) 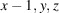; (vi) 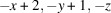.

**Table 2:** Experimental details

Crystal data	
Chemical formula	C_37_H_38_N_6_O_8_·2C_2_H_3_N·2H_2_O
*M*_r_	812.87
Crystal system, space group	Triclinic, *P* 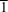
Temperature (K)	100
*a*, *b*, *c* (Å)	9.1493 (2), 12.3058 (3), 20.1432 (5)
α, β, γ (°)	76.076 (2), 84.791 (2), 71.829 (2)
*V* (Å^3^)	2091.19 (10)
*Z*	2
Radiation type	Cu *K*α
μ (mm^−1^)	0.78
Crystal size (mm)	0.9 × 0.3 × 0.05

Data collection	
Diffractometer	Xcalibur, Onyx, Ultra
Absorption correction	Multi-scan (*CrysAlis PRO*, Agilent, 2014)
*T*_min_, *T*_max_	0.728, 1.000
No. of measured, independent and observed [*I* > 2σ(*I*)] reflections	32961, 8593, 7480
*R*_int_	0.048
(sin θ/λ)_max_ (Å^−1^)	0.630

Refinement	
*R*[*F*^2^ > 2σ(*F*^2^)], *wR*(*F*^2^), *S*	0.045, 0.130, 1.04
No. of reflections	8593
No. of parameters	724
H-atom treatment	All H-atom parameters refined
Δρ_max_, Δρ_min_ (e Å^−3^)	0.32, −0.25

Computer programs: *CrysAlis PRO* (Agilent, 2014 ▸), *SHELXT* (Sheldrick, 2015*a* ▸), *SHELXL2013/4* (Sheldrick, 2015*b* ▸) and *OLEX2* (Dolomanov *et al.*, 2009 ▸).

## supplementary crystallographic information

### 4-{[(4-{[1-({[(3,4-Dimethoxyphenyl)methyl](3-acetamidophenyl)carbamoyl}methyl)-1H-1,2,3-triazol-4-yl]methoxy}-3-methoxyphenyl)methyl]amino}benzoic acid–acetonitrile–water (1/2/2) Crystal data

**Table:**

C_37_H_38_N_6_O_8_·2C_2_H_3_N·2H_2_O	*Z* = 2
*M**_r_* = 812.87	*F*(000) = 860
Triclinic, *P*1	*D*_x_ = 1.291 Mg m^−^^3^
*a* = 9.1493 (2) Å	Cu *K*α radiation, λ = 1.54184 Å
*b* = 12.3058 (3) Å	Cell parameters from 15358 reflections
*c* = 20.1432 (5) Å	θ = 3.8–75.8°
α = 76.076 (2)°	µ = 0.78 mm^−^^1^
β = 84.791 (2)°	*T* = 100 K
γ = 71.829 (2)°	Plate, clear colourless
*V* = 2091.19 (10) Å^3^	0.9 × 0.3 × 0.05 mm

### 4-{[(4-{[1-({[(3,4-Dimethoxyphenyl)methyl](3-acetamidophenyl)carbamoyl}methyl)-1H-1,2,3-triazol-4-yl]methoxy}-3-methoxyphenyl)methyl]amino}benzoic acid–acetonitrile–water (1/2/2) Data collection

**Table:**

Xcalibur, Onyx, Ultra diffractometer	7480 reflections with *I* > 2σ(*I*)
Detector resolution: 8.2603 pixels mm^-1^	*R*_int_ = 0.048
ω scans	θ_max_ = 76.4°, θ_min_ = 3.9°
Absorption correction: multi-scan (CrysAlisPro, Agilent, 2014)	*h* = −11→11
*T*_min_ = 0.728, *T*_max_ = 1.000	*k* = −15→15
32961 measured reflections	*l* = −25→25
8593 independent reflections	

### 4-{[(4-{[1-({[(3,4-Dimethoxyphenyl)methyl](3-acetamidophenyl)carbamoyl}methyl)-1H-1,2,3-triazol-4-yl]methoxy}-3-methoxyphenyl)methyl]amino}benzoic acid–acetonitrile–water (1/2/2) Refinement

**Table:**

Refinement on *F*^2^	0 restraints
Least-squares matrix: full	Hydrogen site location: difference Fourier map
*R*[*F*^2^ > 2σ(*F*^2^)] = 0.045	All H-atom parameters refined
*wR*(*F*^2^) = 0.130	*w* = 1/[σ^2^(*F*_o_^2^) + (0.0749*P*)^2^ + 0.6126*P*] where *P* = (*F*_o_^2^ + 2*F*_c_^2^)/3
*S* = 1.04	(Δ/σ)_max_ < 0.001
8593 reflections	Δρ_max_ = 0.32 e Å^−^^3^
724 parameters	Δρ_min_ = −0.25 e Å^−^^3^

### 4-{[(4-{[1-({[(3,4-Dimethoxyphenyl)methyl](3-acetamidophenyl)carbamoyl}methyl)-1H-1,2,3-triazol-4-yl]methoxy}-3-methoxyphenyl)methyl]amino}benzoic acid–acetonitrile–water (1/2/2) Special details

**Table:**

Geometry. All esds (except the esd in the dihedral angle between two l.s. planes) are estimated using the full covariance matrix. The cell esds are taken into account individually in the estimation of esds in distances, angles and torsion angles; correlations between esds in cell parameters are only used when they are defined by crystal symmetry. An approximate (isotropic) treatment of cell esds is used for estimating esds involving l.s. planes.

### 4-{[(4-{[1-({[(3,4-Dimethoxyphenyl)methyl](3-acetamidophenyl)carbamoyl}methyl)-1H-1,2,3-triazol-4-yl]methoxy}-3-methoxyphenyl)methyl]amino}benzoic acid–acetonitrile–water (1/2/2) Fractional atomic coordinates and isotropic or equivalent isotropic displacement parameters (Å^2^)

**Table:**

	*x*	*y*	*z*	*U*_iso_*/*U*_eq_	
O1	1.17136 (13)	0.88775 (10)	−0.09107 (6)	0.0306 (2)	
O2	1.09733 (13)	0.79667 (11)	−0.15897 (6)	0.0352 (3)	
O3	0.27550 (12)	0.47527 (9)	0.04633 (5)	0.0271 (2)	
O4	0.43548 (12)	0.35313 (8)	0.15043 (5)	0.0260 (2)	
O5	0.52857 (11)	−0.19991 (9)	0.30496 (5)	0.0246 (2)	
O6	0.33685 (14)	−0.32839 (10)	0.56423 (6)	0.0334 (2)	
O7	0.19717 (14)	−0.48177 (10)	0.60142 (6)	0.0337 (2)	
O8	−0.13498 (12)	−0.02355 (11)	0.38717 (6)	0.0329 (3)	
O9	−0.00690 (15)	0.13658 (12)	0.29127 (7)	0.0398 (3)	
H9A	−0.045 (3)	0.144 (2)	0.2472 (14)	0.059 (7)*	
H9B	−0.049 (3)	0.074 (3)	0.3171 (15)	0.069 (8)*	
O10	0.76143 (15)	0.36388 (12)	0.29092 (6)	0.0416 (3)	
H10A	0.745 (3)	0.382 (2)	0.3354 (14)	0.060 (7)*	
H10B	0.840 (3)	0.291 (2)	0.2981 (13)	0.055 (7)*	
N1	0.54927 (14)	0.86351 (10)	0.06410 (7)	0.0253 (3)	
H1A	0.548 (2)	0.8990 (19)	0.0983 (11)	0.035 (5)*	
N2	0.57880 (13)	0.09002 (10)	0.17503 (6)	0.0220 (2)	
N3	0.52279 (13)	0.00184 (10)	0.18158 (6)	0.0224 (2)	
N4	0.40357 (12)	0.01865 (10)	0.22590 (6)	0.0211 (2)	
N5	0.33120 (12)	−0.26564 (10)	0.29033 (6)	0.0197 (2)	
N6	−0.22795 (13)	−0.11497 (10)	0.32250 (6)	0.0220 (2)	
H6	−0.309 (2)	−0.1305 (17)	0.3157 (10)	0.030 (5)*	
N7	0.1766 (2)	0.37004 (18)	0.29711 (11)	0.0564 (5)	
N8	0.23890 (19)	−0.01332 (18)	0.40928 (10)	0.0548 (5)	
C1	1.07409 (16)	0.83931 (13)	−0.10892 (7)	0.0249 (3)	
C2	0.94033 (16)	0.84063 (12)	−0.06241 (7)	0.0237 (3)	
C3	0.92719 (17)	0.88189 (12)	−0.00259 (7)	0.0242 (3)	
H3	1.007 (2)	0.9052 (16)	0.0086 (9)	0.027 (4)*	
C4	0.79857 (16)	0.88776 (12)	0.03891 (7)	0.0234 (3)	
H4	0.790 (2)	0.9183 (16)	0.0795 (10)	0.027 (4)*	
C5	0.67743 (16)	0.85103 (11)	0.02278 (7)	0.0218 (3)	
C6	0.69374 (16)	0.80324 (12)	−0.03534 (7)	0.0243 (3)	
H6A	0.618 (2)	0.7733 (16)	−0.0472 (9)	0.024 (4)*	
C7	0.82255 (17)	0.80019 (13)	−0.07722 (7)	0.0248 (3)	
H7	0.833 (2)	0.7680 (16)	−0.1162 (9)	0.025 (4)*	
C8	0.41784 (16)	0.82927 (12)	0.05275 (8)	0.0258 (3)	
H8A	0.396 (2)	0.8517 (17)	0.0036 (11)	0.033 (5)*	
H8B	0.325 (2)	0.8750 (17)	0.0789 (10)	0.033 (5)*	
C9	0.43676 (15)	0.69880 (12)	0.07817 (7)	0.0221 (3)	
C10	0.52712 (15)	0.63117 (12)	0.13346 (7)	0.0225 (3)	
H10	0.590 (2)	0.6636 (16)	0.1549 (9)	0.027 (4)*	
C11	0.53054 (15)	0.51384 (12)	0.16017 (7)	0.0219 (3)	
H11	0.593 (2)	0.4681 (17)	0.1983 (10)	0.028 (4)*	
C12	0.44282 (15)	0.46574 (12)	0.13011 (7)	0.0214 (3)	
C13	0.35354 (15)	0.53324 (12)	0.07247 (7)	0.0219 (3)	
C14	0.35076 (16)	0.64869 (12)	0.04727 (7)	0.0228 (3)	
H14	0.290 (2)	0.6954 (17)	0.0081 (10)	0.028 (4)*	
C15	0.19367 (19)	0.53567 (14)	−0.01514 (8)	0.0301 (3)	
H15A	0.153 (2)	0.4791 (19)	−0.0284 (11)	0.040 (5)*	
H15B	0.262 (2)	0.5605 (18)	−0.0502 (11)	0.036 (5)*	
H15C	0.106 (2)	0.6029 (18)	−0.0067 (10)	0.032 (5)*	
C16	0.51982 (16)	0.27934 (12)	0.20980 (7)	0.0227 (3)	
H16A	0.629 (2)	0.2738 (16)	0.2041 (9)	0.025 (4)*	
H16B	0.480 (2)	0.3104 (16)	0.2517 (10)	0.027 (4)*	
C17	0.49516 (15)	0.16367 (12)	0.21461 (7)	0.0212 (3)	
C18	0.38211 (15)	0.11808 (12)	0.24765 (7)	0.0222 (3)	
H18	0.303 (2)	0.1451 (17)	0.2770 (10)	0.028 (4)*	
C19	0.31026 (15)	−0.06093 (12)	0.24188 (8)	0.0236 (3)	
H19A	0.218 (2)	−0.0224 (18)	0.2680 (10)	0.036 (5)*	
H19B	0.276 (2)	−0.0694 (16)	0.1975 (10)	0.028 (4)*	
C20	0.40083 (14)	−0.18197 (12)	0.28243 (7)	0.0201 (3)	
C21	0.40887 (15)	−0.38716 (12)	0.32711 (7)	0.0223 (3)	
H21A	0.3859 (19)	−0.4391 (15)	0.3010 (9)	0.020 (4)*	
H21B	0.519 (2)	−0.3942 (15)	0.3252 (9)	0.020 (4)*	
C22	0.34860 (15)	−0.41398 (12)	0.39953 (7)	0.0218 (3)	
C23	0.37111 (15)	−0.35337 (12)	0.44665 (7)	0.0235 (3)	
H23	0.425 (2)	−0.2960 (17)	0.4345 (10)	0.031 (5)*	
C24	0.31919 (16)	−0.37859 (12)	0.51336 (8)	0.0252 (3)	
C25	0.24218 (16)	−0.46476 (13)	0.53414 (8)	0.0269 (3)	
C26	0.21904 (17)	−0.52333 (13)	0.48759 (8)	0.0283 (3)	
H26	0.164 (2)	−0.5831 (19)	0.5014 (11)	0.039 (5)*	
C27	0.27249 (16)	−0.49770 (13)	0.42003 (8)	0.0265 (3)	
H27	0.255 (2)	−0.5410 (17)	0.3872 (10)	0.029 (4)*	
C28	0.4173 (2)	−0.24290 (15)	0.54789 (9)	0.0375 (4)	
H28A	0.362 (2)	−0.1763 (19)	0.5127 (11)	0.038 (5)*	
H28B	0.420 (3)	−0.219 (2)	0.5891 (13)	0.052 (6)*	
H28C	0.526 (3)	−0.277 (2)	0.5303 (12)	0.050 (6)*	
C29	0.14031 (19)	−0.57922 (15)	0.62854 (9)	0.0339 (3)	
H29A	0.043 (3)	−0.5705 (19)	0.6095 (11)	0.041 (5)*	
H29B	0.129 (2)	−0.5808 (18)	0.6776 (11)	0.036 (5)*	
H29C	0.221 (2)	−0.6525 (19)	0.6222 (10)	0.035 (5)*	
C30	0.17665 (14)	−0.24101 (11)	0.26830 (7)	0.0190 (3)	
C31	0.15460 (15)	−0.27707 (12)	0.21066 (7)	0.0217 (3)	
H31	0.238 (2)	−0.3187 (16)	0.1868 (9)	0.026 (4)*	
C32	0.00458 (16)	−0.25343 (13)	0.18981 (7)	0.0237 (3)	
H32	−0.013 (2)	−0.2783 (15)	0.1487 (9)	0.023 (4)*	
C33	−0.11947 (15)	−0.19647 (12)	0.22621 (7)	0.0226 (3)	
H33	−0.223 (2)	−0.1797 (15)	0.2128 (9)	0.022 (4)*	
C34	−0.09591 (15)	−0.16253 (11)	0.28466 (7)	0.0198 (3)	
C35	0.05423 (15)	−0.18473 (11)	0.30612 (7)	0.0198 (3)	
H35	0.0685 (18)	−0.1614 (14)	0.3472 (9)	0.017 (4)*	
C36	−0.23841 (16)	−0.05732 (13)	0.37295 (7)	0.0251 (3)	
C37	−0.39062 (19)	−0.03660 (17)	0.41090 (10)	0.0367 (4)	
H37A	−0.449 (3)	−0.081 (2)	0.4053 (12)	0.050 (6)*	
H37B	−0.459 (3)	0.044 (3)	0.3891 (15)	0.074 (8)*	
H37C	−0.378 (3)	−0.026 (3)	0.4552 (15)	0.069 (8)*	
C38	0.0199 (2)	0.43962 (19)	0.18561 (11)	0.0466 (4)	
H38A	−0.021 (3)	0.377 (2)	0.1815 (14)	0.062 (7)*	
H38B	0.089 (3)	0.456 (3)	0.1440 (16)	0.075 (9)*	
H38C	−0.070 (4)	0.508 (3)	0.1896 (15)	0.077 (9)*	
C39	0.1066 (2)	0.40144 (17)	0.24797 (11)	0.0424 (4)	
C40	0.0287 (3)	0.1594 (3)	0.44546 (12)	0.0588 (6)	
H40A	0.065 (3)	0.223 (3)	0.4272 (15)	0.064 (8)*	
H40B	0.035 (4)	0.156 (3)	0.4940 (19)	0.091 (10)*	
H40C	−0.071 (4)	0.163 (3)	0.4316 (18)	0.091 (10)*	
C41	0.1481 (2)	0.06327 (19)	0.42515 (9)	0.0446 (4)	
H1	1.250 (3)	0.887 (2)	−0.1221 (14)	0.064 (7)*	

### 4-{[(4-{[1-({[(3,4-Dimethoxyphenyl)methyl](3-acetamidophenyl)carbamoyl}methyl)-1H-1,2,3-triazol-4-yl]methoxy}-3-methoxyphenyl)methyl]amino}benzoic acid–acetonitrile–water (1/2/2) Atomic displacement parameters (Å^2^)

**Table:**

	*U*^11^	*U*^22^	*U*^33^	*U*^12^	*U*^13^	*U*^23^
O1	0.0311 (5)	0.0353 (6)	0.0329 (6)	−0.0196 (5)	0.0045 (4)	−0.0105 (5)
O2	0.0362 (6)	0.0463 (7)	0.0314 (6)	−0.0201 (5)	0.0051 (4)	−0.0159 (5)
O3	0.0324 (5)	0.0224 (5)	0.0296 (5)	−0.0128 (4)	−0.0083 (4)	−0.0028 (4)
O4	0.0312 (5)	0.0170 (5)	0.0316 (5)	−0.0117 (4)	−0.0086 (4)	0.0003 (4)
O5	0.0168 (4)	0.0234 (5)	0.0345 (5)	−0.0088 (4)	−0.0057 (4)	−0.0025 (4)
O6	0.0450 (6)	0.0288 (6)	0.0304 (5)	−0.0154 (5)	0.0025 (5)	−0.0094 (4)
O7	0.0404 (6)	0.0292 (6)	0.0307 (5)	−0.0144 (5)	0.0049 (5)	−0.0021 (4)
O8	0.0261 (5)	0.0434 (6)	0.0404 (6)	−0.0178 (5)	0.0028 (4)	−0.0218 (5)
O9	0.0431 (7)	0.0417 (7)	0.0372 (6)	−0.0162 (5)	−0.0080 (5)	−0.0063 (5)
O10	0.0418 (7)	0.0474 (7)	0.0309 (6)	−0.0043 (6)	−0.0078 (5)	−0.0091 (5)
N1	0.0298 (6)	0.0197 (6)	0.0297 (6)	−0.0121 (5)	0.0026 (5)	−0.0066 (5)
N2	0.0206 (5)	0.0183 (5)	0.0281 (6)	−0.0088 (4)	−0.0020 (4)	−0.0026 (4)
N3	0.0196 (5)	0.0197 (5)	0.0288 (6)	−0.0082 (4)	−0.0004 (4)	−0.0041 (4)
N4	0.0180 (5)	0.0180 (5)	0.0278 (6)	−0.0080 (4)	−0.0008 (4)	−0.0024 (4)
N5	0.0158 (5)	0.0173 (5)	0.0264 (6)	−0.0065 (4)	−0.0033 (4)	−0.0028 (4)
N6	0.0153 (5)	0.0238 (6)	0.0306 (6)	−0.0093 (4)	−0.0010 (4)	−0.0084 (5)
N7	0.0436 (9)	0.0611 (11)	0.0759 (13)	−0.0141 (8)	0.0043 (9)	−0.0409 (10)
N8	0.0297 (8)	0.0633 (11)	0.0567 (10)	−0.0044 (8)	−0.0040 (7)	0.0026 (9)
C1	0.0266 (7)	0.0235 (7)	0.0256 (7)	−0.0101 (5)	−0.0030 (5)	−0.0029 (5)
C2	0.0254 (6)	0.0209 (6)	0.0252 (7)	−0.0089 (5)	−0.0030 (5)	−0.0025 (5)
C3	0.0272 (7)	0.0204 (6)	0.0279 (7)	−0.0110 (5)	−0.0057 (5)	−0.0034 (5)
C4	0.0298 (7)	0.0195 (6)	0.0231 (6)	−0.0107 (5)	−0.0036 (5)	−0.0034 (5)
C5	0.0258 (6)	0.0142 (6)	0.0242 (6)	−0.0071 (5)	−0.0031 (5)	0.0002 (5)
C6	0.0246 (6)	0.0223 (7)	0.0296 (7)	−0.0103 (5)	−0.0040 (5)	−0.0067 (5)
C7	0.0278 (7)	0.0239 (7)	0.0252 (7)	−0.0097 (5)	−0.0037 (5)	−0.0062 (5)
C8	0.0246 (7)	0.0192 (7)	0.0333 (8)	−0.0086 (5)	0.0012 (6)	−0.0035 (6)
C9	0.0202 (6)	0.0184 (6)	0.0279 (7)	−0.0074 (5)	0.0039 (5)	−0.0048 (5)
C10	0.0199 (6)	0.0204 (6)	0.0294 (7)	−0.0083 (5)	0.0009 (5)	−0.0071 (5)
C11	0.0208 (6)	0.0189 (6)	0.0264 (7)	−0.0071 (5)	−0.0008 (5)	−0.0041 (5)
C12	0.0227 (6)	0.0157 (6)	0.0266 (6)	−0.0084 (5)	0.0009 (5)	−0.0033 (5)
C13	0.0213 (6)	0.0210 (6)	0.0257 (6)	−0.0087 (5)	−0.0002 (5)	−0.0062 (5)
C14	0.0226 (6)	0.0211 (6)	0.0241 (6)	−0.0069 (5)	0.0004 (5)	−0.0037 (5)
C15	0.0338 (8)	0.0310 (8)	0.0286 (7)	−0.0139 (7)	−0.0072 (6)	−0.0043 (6)
C16	0.0228 (6)	0.0178 (6)	0.0279 (7)	−0.0085 (5)	−0.0044 (5)	−0.0015 (5)
C17	0.0201 (6)	0.0179 (6)	0.0259 (6)	−0.0070 (5)	−0.0032 (5)	−0.0025 (5)
C18	0.0209 (6)	0.0174 (6)	0.0274 (7)	−0.0059 (5)	−0.0017 (5)	−0.0029 (5)
C19	0.0175 (6)	0.0200 (6)	0.0335 (7)	−0.0090 (5)	−0.0033 (5)	−0.0010 (5)
C20	0.0166 (6)	0.0202 (6)	0.0244 (6)	−0.0071 (5)	−0.0011 (5)	−0.0045 (5)
C21	0.0184 (6)	0.0175 (6)	0.0300 (7)	−0.0047 (5)	−0.0036 (5)	−0.0034 (5)
C22	0.0176 (6)	0.0175 (6)	0.0282 (7)	−0.0036 (5)	−0.0049 (5)	−0.0017 (5)
C23	0.0224 (6)	0.0173 (6)	0.0307 (7)	−0.0076 (5)	−0.0019 (5)	−0.0024 (5)
C24	0.0249 (6)	0.0200 (6)	0.0302 (7)	−0.0053 (5)	−0.0022 (5)	−0.0058 (5)
C25	0.0246 (7)	0.0226 (7)	0.0290 (7)	−0.0049 (5)	−0.0005 (5)	−0.0004 (5)
C26	0.0264 (7)	0.0243 (7)	0.0350 (8)	−0.0130 (6)	−0.0037 (6)	0.0002 (6)
C27	0.0260 (7)	0.0224 (7)	0.0319 (7)	−0.0092 (5)	−0.0073 (6)	−0.0026 (6)
C28	0.0511 (10)	0.0288 (8)	0.0385 (9)	−0.0171 (7)	−0.0040 (8)	−0.0108 (7)
C29	0.0319 (8)	0.0321 (8)	0.0341 (8)	−0.0131 (7)	0.0004 (6)	0.0034 (6)
C30	0.0166 (6)	0.0162 (6)	0.0242 (6)	−0.0079 (5)	−0.0029 (5)	−0.0001 (5)
C31	0.0196 (6)	0.0212 (6)	0.0241 (6)	−0.0069 (5)	−0.0002 (5)	−0.0042 (5)
C32	0.0232 (6)	0.0259 (7)	0.0236 (6)	−0.0085 (5)	−0.0037 (5)	−0.0059 (5)
C33	0.0179 (6)	0.0241 (7)	0.0273 (7)	−0.0089 (5)	−0.0039 (5)	−0.0043 (5)
C34	0.0172 (6)	0.0178 (6)	0.0249 (6)	−0.0072 (5)	−0.0007 (5)	−0.0028 (5)
C35	0.0187 (6)	0.0188 (6)	0.0238 (6)	−0.0090 (5)	−0.0022 (5)	−0.0034 (5)
C36	0.0216 (6)	0.0272 (7)	0.0293 (7)	−0.0100 (5)	0.0010 (5)	−0.0084 (6)
C37	0.0266 (7)	0.0482 (10)	0.0472 (10)	−0.0189 (7)	0.0108 (7)	−0.0268 (8)
C38	0.0472 (10)	0.0452 (11)	0.0556 (11)	−0.0218 (9)	0.0120 (9)	−0.0208 (9)
C39	0.0365 (9)	0.0405 (10)	0.0609 (12)	−0.0156 (7)	0.0110 (8)	−0.0307 (9)
C40	0.0542 (13)	0.0686 (16)	0.0432 (11)	−0.0025 (11)	−0.0065 (9)	−0.0120 (11)
C41	0.0292 (8)	0.0584 (12)	0.0374 (9)	−0.0112 (8)	−0.0062 (7)	0.0054 (8)

### 4-{[(4-{[1-({[(3,4-Dimethoxyphenyl)methyl](3-acetamidophenyl)carbamoyl}methyl)-1H-1,2,3-triazol-4-yl]methoxy}-3-methoxyphenyl)methyl]amino}benzoic acid–acetonitrile–water (1/2/2) Geometric parameters (Å, º)

**Table:**

O1—C1	1.3300 (17)	C13—C14	1.382 (2)
O1—H1	0.91 (3)	C14—H14	0.961 (19)
O2—C1	1.2178 (18)	C15—H15A	0.98 (2)
O3—C13	1.3648 (16)	C15—H15B	0.96 (2)
O3—C15	1.4228 (18)	C15—H15C	0.99 (2)
O4—C12	1.3683 (16)	C16—H16A	0.975 (19)
O4—C16	1.4347 (17)	C16—H16B	1.002 (19)
O5—C20	1.2280 (16)	C16—C17	1.4887 (19)
O6—C24	1.3619 (18)	C17—C18	1.373 (2)
O6—C28	1.425 (2)	C18—H18	0.922 (19)
O7—C25	1.3661 (19)	C19—H19A	1.00 (2)
O7—C29	1.424 (2)	C19—H19B	1.012 (19)
O8—C36	1.2255 (18)	C19—C20	1.5297 (19)
O9—H9A	0.96 (3)	C21—H21A	0.997 (17)
O9—H9B	0.99 (3)	C21—H21B	0.979 (17)
O10—H10A	0.96 (3)	C21—C22	1.509 (2)
O10—H10B	0.95 (3)	C22—C23	1.4041 (19)
N1—H1A	0.90 (2)	C22—C27	1.381 (2)
N1—C5	1.3650 (19)	C23—H23	0.96 (2)
N1—C8	1.4450 (19)	C23—C24	1.379 (2)
N2—N3	1.3127 (16)	C24—C25	1.412 (2)
N2—C17	1.3590 (18)	C25—C26	1.379 (2)
N3—N4	1.3419 (16)	C26—H26	0.99 (2)
N4—C18	1.3492 (18)	C26—C27	1.400 (2)
N4—C19	1.4530 (17)	C27—H27	0.995 (19)
N5—C20	1.3428 (17)	C28—H28A	0.98 (2)
N5—C21	1.4798 (17)	C28—H28B	0.95 (3)
N5—C30	1.4387 (16)	C28—H28C	1.02 (2)
N6—H6	0.85 (2)	C29—H29A	0.96 (2)
N6—C34	1.4104 (17)	C29—H29B	0.98 (2)
N6—C36	1.3537 (18)	C29—H29C	1.00 (2)
N7—C39	1.147 (3)	C30—C31	1.3900 (19)
N8—C41	1.135 (3)	C30—C35	1.3891 (19)
C1—C2	1.470 (2)	C31—H31	0.940 (19)
C2—C3	1.398 (2)	C31—C32	1.3935 (19)
C2—C7	1.4007 (19)	C32—H32	0.991 (18)
C3—H3	0.926 (19)	C32—C33	1.385 (2)
C3—C4	1.373 (2)	C33—H33	0.955 (18)
C4—H4	0.967 (19)	C33—C34	1.3949 (19)
C4—C5	1.4096 (19)	C34—C35	1.4019 (18)
C5—C6	1.4083 (19)	C35—H35	0.971 (17)
C6—H6A	0.953 (18)	C36—C37	1.506 (2)
C6—C7	1.382 (2)	C37—H37A	0.91 (3)
C7—H7	0.946 (18)	C37—H37B	1.01 (3)
C8—H8A	0.98 (2)	C37—H37C	0.95 (3)
C8—H8B	1.04 (2)	C38—H38A	0.98 (3)
C8—C9	1.5222 (19)	C38—H38B	1.02 (3)
C9—C10	1.382 (2)	C38—H38C	0.99 (3)
C9—C14	1.4010 (19)	C38—C39	1.446 (3)
C10—H10	0.971 (19)	C40—H40A	0.93 (3)
C10—C11	1.4057 (19)	C40—H40B	0.98 (4)
C11—H11	0.95 (2)	C40—H40C	0.96 (4)
C11—C12	1.3851 (19)	C40—C41	1.451 (3)
C12—C13	1.4106 (19)		
			
C1—O1—H1	110.4 (17)	N4—C19—H19B	108.4 (11)
C13—O3—C15	117.34 (11)	N4—C19—C20	111.40 (11)
C12—O4—C16	117.77 (10)	H19A—C19—H19B	109.4 (15)
C24—O6—C28	117.66 (12)	C20—C19—H19A	112.2 (12)
C25—O7—C29	117.23 (13)	C20—C19—H19B	109.1 (11)
H9A—O9—H9B	98 (2)	O5—C20—N5	123.36 (12)
H10A—O10—H10B	103 (2)	O5—C20—C19	121.54 (12)
C5—N1—H1A	115.9 (13)	N5—C20—C19	115.09 (11)
C5—N1—C8	123.62 (12)	N5—C21—H21A	105.7 (10)
C8—N1—H1A	120.4 (13)	N5—C21—H21B	105.6 (10)
N3—N2—C17	109.57 (11)	N5—C21—C22	112.09 (11)
N2—N3—N4	106.74 (11)	H21A—C21—H21B	111.7 (14)
N3—N4—C18	111.20 (11)	C22—C21—H21A	109.2 (10)
N3—N4—C19	120.82 (11)	C22—C21—H21B	112.4 (10)
C18—N4—C19	127.87 (12)	C23—C22—C21	119.45 (12)
C20—N5—C21	120.23 (11)	C27—C22—C21	120.83 (13)
C20—N5—C30	122.53 (11)	C27—C22—C23	119.72 (13)
C30—N5—C21	117.11 (10)	C22—C23—H23	122.0 (11)
C34—N6—H6	115.9 (13)	C24—C23—C22	120.06 (13)
C36—N6—H6	115.8 (13)	C24—C23—H23	117.9 (11)
C36—N6—C34	128.01 (12)	O6—C24—C23	125.84 (13)
O1—C1—C2	113.95 (12)	O6—C24—C25	114.18 (13)
O2—C1—O1	121.94 (13)	C23—C24—C25	119.97 (13)
O2—C1—C2	124.10 (13)	O7—C25—C24	114.25 (13)
C3—C2—C1	121.20 (13)	O7—C25—C26	125.94 (14)
C3—C2—C7	118.35 (13)	C26—C25—C24	119.81 (14)
C7—C2—C1	120.45 (13)	C25—C26—H26	120.5 (12)
C2—C3—H3	118.7 (11)	C25—C26—C27	119.96 (14)
C4—C3—C2	120.75 (13)	C27—C26—H26	119.5 (12)
C4—C3—H3	120.5 (11)	C22—C27—C26	120.47 (13)
C3—C4—H4	119.9 (11)	C22—C27—H27	120.8 (11)
C3—C4—C5	120.93 (13)	C26—C27—H27	118.7 (11)
C5—C4—H4	119.2 (11)	O6—C28—H28A	109.8 (12)
N1—C5—C4	118.57 (12)	O6—C28—H28B	106.2 (15)
N1—C5—C6	122.84 (13)	O6—C28—H28C	111.1 (14)
C6—C5—C4	118.58 (13)	H28A—C28—H28B	110.3 (19)
C5—C6—H6A	121.9 (10)	H28A—C28—H28C	108.8 (18)
C7—C6—C5	119.59 (13)	H28B—C28—H28C	110.6 (19)
C7—C6—H6A	118.5 (10)	O7—C29—H29A	113.0 (13)
C2—C7—H7	119.2 (11)	O7—C29—H29B	103.1 (12)
C6—C7—C2	121.65 (13)	O7—C29—H29C	108.7 (12)
C6—C7—H7	119.2 (11)	H29A—C29—H29B	111.0 (17)
N1—C8—H8A	109.6 (12)	H29A—C29—H29C	112.3 (17)
N1—C8—H8B	106.9 (11)	H29B—C29—H29C	108.2 (17)
N1—C8—C9	114.37 (12)	C31—C30—N5	118.76 (12)
H8A—C8—H8B	109.2 (16)	C35—C30—N5	119.15 (11)
C9—C8—H8A	108.6 (12)	C35—C30—C31	122.07 (12)
C9—C8—H8B	108.1 (11)	C30—C31—H31	121.3 (11)
C10—C9—C8	121.97 (13)	C30—C31—C32	118.46 (12)
C10—C9—C14	119.47 (12)	C32—C31—H31	120.2 (11)
C14—C9—C8	118.39 (12)	C31—C32—H32	119.4 (10)
C9—C10—H10	120.8 (11)	C33—C32—C31	120.63 (12)
C9—C10—C11	120.82 (13)	C33—C32—H32	119.9 (10)
C11—C10—H10	118.4 (11)	C32—C33—H33	121.9 (10)
C10—C11—H11	120.3 (11)	C32—C33—C34	120.35 (12)
C12—C11—C10	119.28 (13)	C34—C33—H33	117.8 (10)
C12—C11—H11	120.4 (11)	C33—C34—N6	116.94 (11)
O4—C12—C11	125.59 (12)	C33—C34—C35	119.84 (12)
O4—C12—C13	114.17 (12)	C35—C34—N6	123.03 (12)
C11—C12—C13	120.23 (12)	C30—C35—C34	118.63 (12)
O3—C13—C12	114.78 (12)	C30—C35—H35	122.6 (10)
O3—C13—C14	125.58 (13)	C34—C35—H35	118.8 (10)
C14—C13—C12	119.64 (12)	O8—C36—N6	124.00 (13)
C9—C14—H14	119.3 (11)	O8—C36—C37	121.88 (13)
C13—C14—C9	120.53 (13)	N6—C36—C37	114.12 (12)
C13—C14—H14	120.1 (11)	C36—C37—H37A	115.1 (15)
O3—C15—H15A	106.1 (13)	C36—C37—H37B	108.2 (17)
O3—C15—H15B	110.2 (12)	C36—C37—H37C	109.4 (17)
O3—C15—H15C	110.2 (11)	H37A—C37—H37B	100 (2)
H15A—C15—H15B	109.8 (17)	H37A—C37—H37C	121 (2)
H15A—C15—H15C	108.7 (17)	H37B—C37—H37C	101 (2)
H15B—C15—H15C	111.6 (17)	H38A—C38—H38B	109 (2)
O4—C16—H16A	111.0 (11)	H38A—C38—H38C	106 (2)
O4—C16—H16B	111.4 (11)	H38B—C38—H38C	114 (2)
O4—C16—C17	103.73 (10)	C39—C38—H38A	108.4 (16)
H16A—C16—H16B	107.9 (15)	C39—C38—H38B	110.6 (16)
C17—C16—H16A	111.3 (11)	C39—C38—H38C	108.8 (18)
C17—C16—H16B	111.5 (11)	N7—C39—C38	179.1 (2)
N2—C17—C16	120.91 (12)	H40A—C40—H40B	99 (3)
N2—C17—C18	107.84 (12)	H40A—C40—H40C	118 (3)
C18—C17—C16	130.64 (13)	H40B—C40—H40C	114 (3)
N4—C18—C17	104.64 (12)	C41—C40—H40A	102.5 (17)
N4—C18—H18	123.2 (12)	C41—C40—H40B	111 (2)
C17—C18—H18	132.1 (12)	C41—C40—H40C	111 (2)
N4—C19—H19A	106.2 (12)	N8—C41—C40	178.3 (2)
			
O1—C1—C2—C3	−4.54 (19)	C11—C12—C13—C14	−1.8 (2)
O1—C1—C2—C7	175.50 (13)	C12—O4—C16—C17	−176.54 (11)
O2—C1—C2—C3	174.42 (14)	C12—C13—C14—C9	0.5 (2)
O2—C1—C2—C7	−5.5 (2)	C14—C9—C10—C11	−1.7 (2)
O3—C13—C14—C9	−179.55 (12)	C15—O3—C13—C12	−174.93 (12)
O4—C12—C13—O3	−0.33 (17)	C15—O3—C13—C14	5.1 (2)
O4—C12—C13—C14	179.62 (12)	C16—O4—C12—C11	3.0 (2)
O4—C16—C17—N2	80.36 (15)	C16—O4—C12—C13	−178.42 (12)
O4—C16—C17—C18	−89.54 (17)	C16—C17—C18—N4	170.74 (13)
O6—C24—C25—O7	0.75 (18)	C17—N2—N3—N4	−0.44 (14)
O6—C24—C25—C26	−179.07 (13)	C18—N4—C19—C20	−115.62 (15)
O7—C25—C26—C27	−179.49 (14)	C19—N4—C18—C17	−176.24 (12)
N1—C5—C6—C7	175.90 (13)	C20—N5—C21—C22	101.22 (14)
N1—C8—C9—C10	29.93 (19)	C20—N5—C30—C31	107.10 (15)
N1—C8—C9—C14	−154.72 (13)	C20—N5—C30—C35	−74.49 (17)
N2—N3—N4—C18	0.34 (15)	C21—N5—C20—O5	−0.7 (2)
N2—N3—N4—C19	176.79 (11)	C21—N5—C20—C19	178.47 (12)
N2—C17—C18—N4	−0.16 (15)	C21—N5—C30—C31	−77.15 (16)
N3—N2—C17—C16	−171.58 (11)	C21—N5—C30—C35	101.26 (14)
N3—N2—C17—C18	0.38 (15)	C21—C22—C23—C24	−178.77 (12)
N3—N4—C18—C17	−0.11 (15)	C21—C22—C27—C26	179.01 (13)
N3—N4—C19—C20	68.58 (16)	C22—C23—C24—O6	178.35 (13)
N4—C19—C20—O5	8.79 (19)	C22—C23—C24—C25	−0.5 (2)
N4—C19—C20—N5	−170.36 (11)	C23—C22—C27—C26	−0.6 (2)
N5—C21—C22—C23	−64.78 (15)	C23—C24—C25—O7	179.76 (12)
N5—C21—C22—C27	115.57 (13)	C23—C24—C25—C26	−0.1 (2)
N5—C30—C31—C32	179.70 (12)	C24—C25—C26—C27	0.3 (2)
N5—C30—C35—C34	−179.20 (11)	C25—C26—C27—C22	0.0 (2)
N6—C34—C35—C30	174.52 (12)	C27—C22—C23—C24	0.9 (2)
C1—C2—C3—C4	177.01 (13)	C28—O6—C24—C23	−0.7 (2)
C1—C2—C7—C6	−178.31 (13)	C28—O6—C24—C25	178.27 (13)
C2—C3—C4—C5	0.8 (2)	C29—O7—C25—C24	−170.61 (13)
C3—C2—C7—C6	1.7 (2)	C29—O7—C25—C26	9.2 (2)
C3—C4—C5—N1	−177.16 (13)	C30—N5—C20—O5	174.96 (12)
C3—C4—C5—C6	2.7 (2)	C30—N5—C20—C19	−5.91 (18)
C4—C5—C6—C7	−4.0 (2)	C30—N5—C21—C22	−74.63 (14)
C5—N1—C8—C9	80.99 (17)	C30—C31—C32—C33	−0.7 (2)
C5—C6—C7—C2	1.8 (2)	C31—C30—C35—C34	−0.8 (2)
C7—C2—C3—C4	−3.0 (2)	C31—C32—C33—C34	−0.4 (2)
C8—N1—C5—C4	179.92 (13)	C32—C33—C34—N6	−174.22 (13)
C8—N1—C5—C6	0.0 (2)	C32—C33—C34—C35	0.9 (2)
C8—C9—C10—C11	173.58 (12)	C33—C34—C35—C30	−0.28 (19)
C8—C9—C14—C13	−174.25 (12)	C34—N6—C36—O8	10.5 (2)
C9—C10—C11—C12	0.5 (2)	C34—N6—C36—C37	−169.90 (14)
C10—C9—C14—C13	1.2 (2)	C35—C30—C31—C32	1.3 (2)
C10—C11—C12—O4	179.72 (13)	C36—N6—C34—C33	−167.21 (14)
C10—C11—C12—C13	1.3 (2)	C36—N6—C34—C35	17.8 (2)
C11—C12—C13—O3	178.29 (12)		

### 4-{[(4-{[1-({[(3,4-Dimethoxyphenyl)methyl](3-acetamidophenyl)carbamoyl}methyl)-1H-1,2,3-triazol-4-yl]methoxy}-3-methoxyphenyl)methyl]amino}benzoic acid–acetonitrile–water (1/2/2) Hydrogen-bond geometry (Å, º)

**Table:**

*D*—H···*A*	*D*—H	H···*A*	*D*···*A*	*D*—H···*A*
O9—H9*A*···O2^i^	0.96 (3)	1.80 (3)	2.7173 (17)	160 (2)
O9—H9*B*···O8	0.99 (3)	1.90 (3)	2.8610 (18)	164 (2)
O10—H10*A*···O6^ii^	0.96 (3)	2.12 (3)	2.9441 (17)	144 (2)
O10—H10*A*···O7^ii^	0.96 (3)	2.17 (3)	2.9928 (17)	143 (2)
O10—H10*B*···O9^iii^	0.95 (3)	2.00 (3)	2.9346 (19)	167 (2)
N1—H1*A*···N3^iv^	0.90 (2)	2.29 (2)	3.1834 (17)	174.6 (18)
N6—H6···O5^v^	0.85 (2)	1.98 (2)	2.8200 (15)	168.6 (19)
O1—H1···N2^vi^	0.91 (3)	1.87 (3)	2.7614 (16)	166 (3)

Symmetry codes: (i) −*x*+1, −*y*+1, −*z*; (ii) −*x*+1, −*y*, −*z*+1; (iii) *x*+1, *y*, *z*; (iv) *x*, *y*+1, *z*; (v) *x*−1, *y*, *z*; (vi) −*x*+2, −*y*+1, −*z*.
