# Supplementary material for: Binary solvent participation in crystals of a multi-aromatic 1,2,3-triazole
Source: Acta Crystallogr E Crystallogr Commun. 2025 Jan 1;81(Pt 1):38–41. doi: 10.1107/S2056989024011915 (PMC11701773; doi:10.1107/S2056989024011915)
Supplement: Supplementary file 3 [file e-81-00038-sup3.doc]

Supplementary Information for:

“Binary solvent participation in the crystallization of a multi-aromatic 1,2,3-triazole”

Acta Crystallographica E, Sep 2024

All reagents were obtained from Aldrich. Reductive aminations were performed according to Touchette (Touchette, K.M. 2006).

Alkyne (3 steps):

1. *3-Methoxy-4-propargyloxybenzaldehyde:* A 100 ml rb flask is charged with 5.0 g (32.9 mmole) vanillin and 5.1 g (37.0mmole) K2CO3. The solids are mixed together to form a uniform powder before 5.7 ml (36.3 mmole) propargyl benzenesulfonate is added via pipette. The dark paste is mixed with a spatula briefly before it is heated to 373 K for 1 hr. (a little effervescence occurs at about 353 K, the reaction was protected from the atmosphere with N2). During the cooling period, the hard solid should be stirred once or twice to facilitate its removal from the flask. The solid is ground to a fine paste with a little water in a mortar and pestle before it is stirred in 100 ml water for 30 m, vacuum filtered, and washed well with water. After air drying overnight, the tan solid is boiled in 20 ml ethanol, filtered, and allowed to stand to give 5.1 g (82%) dark amber crystals. A second crystallization from 20 ml toluene (filtering unnecessary) gave 4.3 g (69%) of material with no improvement in color, mp 85-86. ^1^H NMR (400 MHz, CDCl_3_): 2.58 (t, 1H, *J* = 2.4 Hz), 3.95 (s, 3H), 4.87 (d, 2H, *J* = 2.4 Hz), 6.98-7.48 (m, 3H), 9.88 (s, 1H).

2. *Ethyl 4-(4-propargyloxy-3-methoxyphenyl)methylaminobenzoate:* A 125 ml Erlenmeyer flask is charged with 1.65 g (10.0 mmole) ethyl 4-aminobenzoate and 1.90 g (10.0 mmole) propargyl vanillin. The contents of the flask are stirred and heated at 373 K for 1 h before being cooled. The imine is treated with 0.5 g (13.2 mmole) NaBH4 and 30 ml 95% ethanol and heated in a water bath at (323-333 K) for 1 h. The solid imine should come dislodged from the bottom of the flask during this time. The mixture is poured into 100 ml water, followed by 6 M HCl, which is added ¼ ml at a time until foaming ceases, and the final pH is 2-3. The white precipitate is collected by vacuum filtration and washed well with water. Recrystallization from 20 ml EtOH and 5-10 ml acetone gives flakey light tan crystals, about 2.3 g (68%) after 2-3 recrystallizations, mp 114-115. HPLC analysis should show minimal imine at this point.

3. *4-(4-Propargyloxy-3-methoxyphenyl)methylaminobenzoic acid:* A 2.4 g sample of ethyl 4-(4-propargyloxy-3-methoxyphenyl)methylaminobenzoate (7.1 mmole) is placed in a 250 ml round bottomed flask and treated with 20 ml 1 M NaOH, 20 ml 3% H_2_O_2_, and 40 ml ethanol. The mixture is refluxed 3 h, at which point the clear yellow solution is poured into 80 ml water. The mixture is filtered, and the filtrate is acidified with 25 ml 1 M HCl to pH 1-2. The white precipitate is stirred 1 h and then vacuum filtered to give a white filter cake which weighed 2.1 g (96%) after drying overnight. ^1^H NMR (400 MHz, D_2_O): 3.62 (s, 3H), 4.04 (s, 2H), 4.49 (s, 2H), 6.56-7.66 (m, 7H). ^13^C NMR (D_2_O): 46.4, 55.5, 56.4, 78.0, 111.2, 112.6, 113.7, 119.7, 124.8, 131.0, 133.6, 144.7, 148.6, 150.7, 163.5, 175.5.

Azide (4 steps):

1. *3-Acetamido-N-(3,4-dimethoxybenzylidene)aniline:* A 250 ml beaker is charged with 3.1 g (20.6 mmole) 3’-aminoacetanilide and 3.4 g (20.5 mmole) 3,4-dimethoxybenzaldehyde. The contents of the beaker are melted and swirled together on a hot plate 5-10 m at 373-423 ºC, some bubbling occurs, and then the brown oil solidifies. The solid is dislodged from the beaker as it cools, ground to a fine powder, and boiled in 20 ml EtOH plus 140-160 ml acetone. The clear solution is boiled to 125 ml, cooled, and the small white plates are collected by vacuum filtration, 4.8 g (78%).

2. *3-Acetamido-N-(3,4-dimethoxybenzyl)aniline:* The purified imine is treated with 0.65 g NaBH_4_ (17.2 mmole), 40 ml 95% ethanol and stirred overnight at room temperature. This mixture is poured into 100 ml water and treated with 2-3 g solid NaHCO_3_ before being stirred 2-3 h, vacuum filtered, and air dried overnight, 4.7 g. The white solid is boiled with 10 ml EtOH and 130 ml acetone, filtered, boiled down to 75 ml, and allowed to crystallize, 3.5 g (58%), mp 158-161. ^1^H NMR (CDCl_3_, 400 MHz): 2.13 (s, 3H), 3.86 (s, 3H), 3.87 (s, 3H), 4.04 (bs, 1H), 4.23(bs, 2H), 6.37 (m, 1H), 6.66 (m, 1H), 6.83 (m, 1H), 6.89 (m, 2H), 7.08 (m, 2H), 7.28 (bs, 1H). ^13^C NMR (CDCl_3_): 24.7, 48.1, 55.8, 55.9, 104.4, 108.7, 108.8, 110.7, 111.1, 119.7, 129.6, 131.7, 138.9, 148.2, 148.9, 149.1, 168.3.

3. *N-(3-Acetylaminophenyl)-N-(3,4-dimethoxybenzyl)-chloroacetamide::* A 2.5 g sample (8.3 mmole) of 3-acetamido-N-(3,4-dimethoxybenzyl)aniline is suspended in 40 ml acetone and treated with 2 g NaHCO_3_ followed by 0.8 ml (9.9 mmole) chloroacetyl chloride. The mixture turns yellow, and then fades to white over 1 h. The suspension is poured into 100 ml water and stirred 1-2 h. Vacuum filtration, followed by air drying, gives 3.1 g white powder, (100%), mp 179-180.

4. N*-(3-Acetamidophenyl)-N-(3,4-dimethoxybenzyl)-azidoacetamide:* A 100 ml round bottomed flask is charged with 3.1 g (8.2 mmole) N-(3-acetylaminophenyl)-N-(3,4-dimethoxybenzyl)chloroacetamide, 0.8 g (12.3 mmole) sodium azide, 120 mg (0.8 mmole) sodium iodide, 30 mg (0.08 mmole) dibenzo-18-crown-6 and 40 ml acetone. The flask is stirred and refluxed overnight, resulting in a heterogeneous mixture. The contents of the flask are cooled and poured into 100 ml water and stirred 1 h, resulting in a granular white precipitate., which is collected by vacuum filtration and air dried overnight to give 3.1 g (100%) white powder, mp 172-173 dec. All azides should be stored in a refrigerator.

References

Touchette, K.M. (2006) *J. Chem. Educ*. **83** 929.
